# “Change Doesn’t Happen by Itself”: A Thematic Analysis of First-Level Leaders’ Experiences Participating in the Leadership and Organizational Change for Implementation (LOCI) Strategy

**DOI:** 10.1007/s10488-022-01199-x

**Published:** 2022-05-18

**Authors:** Randi Hovden Borge, Karina M. Egeland, Gregory A. Aarons, Mark G. Ehrhart, Marisa Sklar, Ane-Marthe Solheim Skar

**Affiliations:** 1 Norwegian Centre for Violence and Traumatic Stress Studies (NKVTS), Gullhaugveien 1, 0484 Oslo, Norway; 2 Department of Psychiatry, University of California, San Diego, 9500 Gilman Drive (0812), La Jolla, CA 92093-0812 USA; 3 UC San Diego ACTRI Dissemination and Implementation Science Center, 9500 Gilman Drive, La Jolla, CA 92093 USA; 4 Child and Adolescent Services Research Center, 3665 Kearny Villa Rd., Suite 200N, San Diego, CA 92123 USA; 5 Department of Psychology, University of Central Florida, 4111 Pictor Lane, Orlando, FL 32816-1390 USA

**Keywords:** Evidence-based practices, Implementation leadership, Implementation strategy, LOCI, Mental health services

## Abstract

The Leadership and Organizational Change for Implementation (LOCI) strategy is a multifaceted implementation strategy that aims to support successful evidence-based practice (EBP) implementation by fostering effective general leadership, implementation leadership, and implementation climate. How implementation strategies are experienced by participants is important for their utilization and effectiveness in supporting EBP implementation. The current study is the first in-depth qualitative study exploring first-level leaders’ experiences of participating in the LOCI strategy. Data were collected as part of a trial where Norwegian child and adult mental health outpatient clinics implemented EBPs for posttraumatic stress disorder (PTSD). Eleven first-level leaders from adult and child clinics participated in semi-structured interviews after completing the LOCI strategy. Data were analyzed through reflexive thematic analysis. The analysis generated four themes related to leaders’ experiences of participating in the LOCI strategy: (1) structuring the EBP implementation, (2) taking responsibility for the EBP implementation, (3) interacting with others about the EBP implementation, and (4) becoming aware of EBP implementation and their own leadership. Most participants experienced the LOCI strategy as beneficial for implementing EBPs for PTSD in their clinic. The strategy succeeded in raising awareness of leadership for EBP implementation, and simultaneously provided participants with tools and support for leading the implementation in their clinic. Two participants experienced LOCI as less beneficial than the others. Our results support the strategy’s potential to engage and empower first-level leaders to get involved in implementation processes and point to important challenges for future research on implementation strategies.

## Background

Implementing practice change in mental health services is challenging, and evidence-based practice (EBP) implementation is likely to fail in the absence of clear and effective strategies. Interest in implementation strategies within implementation science is increasing (Powell et al., 2015; Waltz et al., 2019), including initiatives focusing on implementation leadership (Aarons et al., 2015; Proctor et al., 2019). Previous research indicates that leadership may play a pivotal role in EBP implementation success in various ways, from inspiring and motivating employees to developing new routines and procedures (Meza et al., 2021; Reichenpfader et al., 2015). Yet, many leaders in mental health organizations, particularly first-level leaders (i.e., those who directly supervise direct service staff), generally do not have formal education in leadership or change management (e.g., Falender, 2018; Spehar et al., 2012). Considering their unique position to support EBP implementation through direct access and close contact with practitioners, interventions aimed at training first-level leaders in general leadership and strategic implementation leadership could improve implementation effectiveness.

The Leadership and Organizational Change for Implementation (LOCI) strategy is an implementation strategy that aims to foster general leadership and implementation leadership among first-level leaders while simultaneously engaging middle and upper-level management to create a supportive implementation climate for EBPs in the organization (Aarons et al., 2015, 2017a, 2017b). Viewing first-level leaders as primary change agents in EBP implementation, the strategy places a strong emphasis on leadership development, including individual leadership training and coaching. The program is based on both the full-range leadership model (Bass & Avolio, 1990) and the implementation leadership literature (Aarons et al., 2015), and consists of various components—such as 360-degree feedback, spaced training sessions, and multiple delivery methods (e.g., workshops, coaching)—found to be effective design and delivery features of leadership training programs in general (Lacerenza et al., 2017). Thus, in sum, the LOCI strategy is both multilevel and multifaceted in nature, and targets both development of organizational strategies to support effective EBP implementation and training of first-level leaders in generic and implementation-specific leadership skills.

A mixed-method pilot study from the US involving 12 mental health care leaders and 100 practitioners found that the LOCI strategy was feasible, acceptable, and useful for its participants and led to better leader knowledge, engagement, and change in leadership behavior to support EBPs (Aarons et al., 2015). Qualitative results based on open-ended survey questions and a focus group interview showed that the leaders found the different components of the strategy desirable, practical, and valuable for them in their day-to-day work (Aarons et al., 2015). The LOCI strategy has also been tested quantitatively in Norway in a stepped-wedge study with 47 first-level leaders and 804 therapists during a national implementation of evidence-based practices for posttraumatic stress disorder (EBPs for PTSD) (Egeland et al., 2019a, 2019b). Results demonstrated that when the leaders enrolled in the LOCI strategy, there were significant increases in therapists’ reports of general and implementation leadership, as well as improvement in perceptions of implementation climate (Skar et al., 2022).

Qualitative studies with an in-depth approach to understanding leaders’ experiences of participating in implementation strategies such as the LOCI strategy may yield further insight into how these strategies facilitate learning among participants and are utilized in EBP implementation in practice (Hamilton & Finley, 2019). EBP implementation in mental health services is a complex and dynamic process. Learning to lead in such environments is not straightforward; it involves not only applying newly developed skills, but also less tangible competencies such as the ability to adapt one’s approach to changing circumstances (King & Nesbit, 2015). This implies that implementation strategy evaluations should make use of in-depth qualitative analysis in an attempt to capture these intangible and complex learning outcomes. Furthermore, knowing more about how different participants experience different components of a multifaceted or blended implementation strategy is important, as context and individual characteristics are found to be central for understanding training transfer in leadership development (e.g., Franke & Felfe, 2012; Velada et al., 2007). Tailoring and adapting implementation strategies to support utilization across various contexts—and for participants with different characteristics and varying degrees of engagement—is key to increasing the potential impact of such strategies on implementation success.

As one of the first qualitative studies to investigate implementation strategies targeting implementation leadership, the current study explored how first-level leaders in Norwegian mental health services experienced participating in the LOCI strategy during the implementation of EBPs for PTSD using Braun and Clarke’s (2006) approach to qualitative thematic analysis.

## Methods

### Study Setting

The implementation project was managed by the Norwegian Centre for Violence and Traumatic Stress Studies (NKVTS) and took place in Norwegian child and adult mental health outpatient clinics (Egeland et al., 2019a, 2019b). Data were collected as part of a larger stepped-wedge implementation study where 47 first-level leaders were randomized into three cohorts with each cohort sequentially included in the LOCI strategy at three different time points: in September 2018, January 2019, and May 2019 (please see the study protocol for more information on recruitment of clinics; Egeland et al., 2019a, 2019b). All participating therapists received training in routine screening for exposure to potential traumatizing events and posttraumatic stress symptoms (PTSS), and a sub-sample received training in EBPs for PTSD treatment (trauma-focused cognitive behavioral therapy (TF-CBT) (Cohen et al., 2017) in child clinics and cognitive therapy for PTSD (CT-PTSD) (Ehlers & Clark, 2000) or eye movement desensitization and reprocessing (EMDR) (Shapiro, 1989) in adult clinics). The project, including the current study, was approved by the Norwegian Centre for Research Data (60036/3/LH and 60059/3/OOS).

### The LOCI Strategy

A research team at NKVTS conducted the LOCI trainings, leadership coaching, and organizational strategy activities in Norwegian. A train-the-trainer approach was used where the US based LOCI developers worked closely with the team by assisting with translation and adaptation of training materials and measures, meeting remotely to discuss LOCI components and training approaches, and participating in the in-person training held at NKVTS in Norway. The LOCI developers then observed and gave feedback on trainings, leadership coaching, and organization strategy meetings.

The LOCI strategy was structured to be consistent with the original US-based version (Aarons et al., 2017a, 2017b). For first-level leaders, the initial leadership development training included a two-day interactive workshop with theory on the full-range leadership model (Bass & Avolio, 1990), implementation leadership (Aarons et al., 2014a, 2014b), and implementation climate (Ehrhart et al., 2014; Weiner et al., 2011), supplemented with group discussions and interactive activities (e.g., identifying characteristics of effective leaders). The first-level leaders received individual feedback on leadership and implementation climate based on data from a 360-degree assessment completed by therapists, the first-level leader, and the executive leader in the health trust. Continued leadership development training included quarterly one-day follow-up trainings complemented with 360-degree assessments and feedback.

Throughout the duration of LOCI participation, the first-level leaders participated in weekly consultation calls with a LOCI coach to develop individual leadership development plans. Once a month, the coaching calls were replaced with a group collaboration call for all first-level leaders in the same cohort in order to share experiences and develop an informal learning collaborative. In addition, the first-level leaders and executive leaders within the same health trust participated in quarterly organizational strategy meetings where data on implementation climate were presented, and an overall organizational strategy plan for implementation climate development was created. Once a month, executive leaders participated in a consultation call with a LOCI coach to follow up on the organizational strategy plan.

Individual leadership plans and organizational strategy plans were tailored and adapted throughout the one-year program based on the 360-degree assessment feedback reports and individual experiences and needs of each clinic and health trust through a co-creation process approach involving leaders and LOCI coaches. Although participants were free to decide the content of their own plans, they were advised to include targets that are known to be important for EBP implementation (e.g., routines for systematic trauma screening) and goals that addressed specific issues identified in the results of the 360-degree assessment feedback reports. The individual leadership plans differed both in content and scope as a function of each first-level leader’s focus, motivation, and circumstances. Most leaders chose to include both goals for developing their own generic leadership skills and goals more directly and explicitly connected to the EBP implementation. The two often went hand in hand. For example, leaders practiced their individualized consideration and inspirational motivation skills (i.e., transformational leadership) in supporting and motivating the therapists to start using the EBPs (i.e., supportive implementation leadership).

### Participants and Procedure

**Fig. 1:**
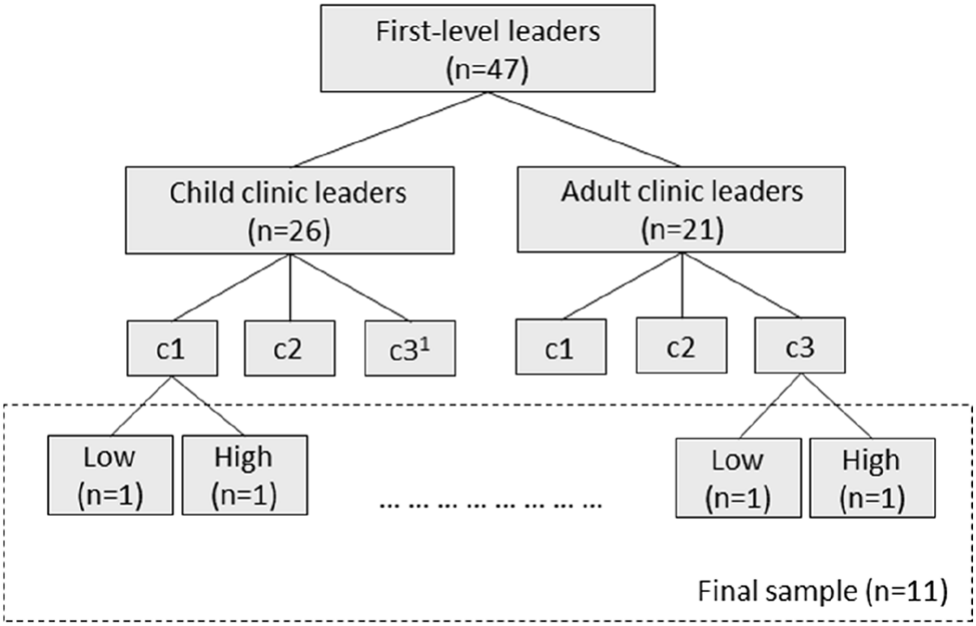
Purposeful sampling approach. ^1^Not able to recruit

After completing LOCI, a sub-sample of leaders (n = 11) participated in qualitative semi-structured interviews about their experiences of the LOCI strategy. Findings from leadership development evaluations suggest that context and participant characteristics such as motivation are important for training transfer (e.g., Franke & Felfe, 2012). To capture the potential variability in leaders’ experiences of the LOCI strategy we used a three-level purposeful sampling approach based on clinic (adult and child), cohort (1–3), and engagement rate (high–low) strategy (see Fig. 1). Thus, in each cohort the leader in both child and adult clinics with the highest and the lowest engagement rate was asked to participate. Engagement rate was based on the sum of training workshops and individual consultation calls each leader had attended. In cases where leaders had the same engagement rate, the number of times attending group consultation calls were used to determine which leaders should first be approached. In cases where the first leader declined, the leader with the second highest (or lowest) engagement rate was approached. Potential participants were contacted by email or telephone by their respective LOCI coaches. Participation was based on voluntary and informed consent and the participants did not receive any remuneration for participating.

The final sample consisted of 11 first-level leaders—five from the child clinics and six from the adult clinics (Fig. 1). We attempted to recruit a sixth leader from the child clinics (cohort 3, low engagement rate), but all declined participation due to reasons related to the COVID-19 pandemic. The participants were seven women and four men; mean age was 49 years (SD = 9.13). Eight leaders were medical doctors or psychologists by training. The remaining three had educational backgrounds in social work. Average years of work experience in current profession was 18 years (SD = 6.65), and, on average, the leaders had occupied their current leadership position for 5.25 years (SD = 3.86).

One master’s student in public management and leadership and one clinical psychologist, both unaffiliated with the LOCI team, conducted the interviews, which lasted approximately one hour each (one face-to-face and the rest by telephone or video conference). The interviews were semi-structured with a pre-defined interview guide based on the different elements of the LOCI strategy (Table 1). The interviewers began by informing participants of their position relative to the LOCI team (i.e., not part of the LOCI team). The study was framed to participants as a way for the project to learn and improve since it was the first time the LOCI strategy had been carried out in this specific context. The participants were encouraged to convey any feedback they had—both positive and negative—as a way to enhance the potential tailoring, adaptation, and benefit of the LOCI strategy for future participants. At the end of the interview, participants were asked if there was anything they wanted to add to what they had already conveyed. The interviews were audio-recorded and stored on a secure digital platform for research data. The interviews were orthographically transcribed using regular word processing software.

**Table 1:** Overview of interview guide

Topic	Example questions
Prior to implementation	How much experience do you have with implementation? Were you involved in the decision to implement EBPs for PTSD in your clinic?
Overall impression	Can you tell me about your overall impression of LOCI? Were some of the components more useful than others?
Data feedback reports	How was it to receive feedback on your leadership behavior?
Individual consultations	What was useful about the individual consultations?
Cooperation with executive leader	Can you tell me about the cooperation between the executive leader and yourself before and after LOCI?
Participating as part of a group	How was it to work together in a group with other leaders?
Role of LOCI in the EBP implementation	What has been the most challenging in this EBP implementation? How have you used LOCI in your work?
Future implementations	Do you feel better prepared to lead other change processes and implementation projects after LOCI?
Practical considerations	If we were to start a new LOCI intervention, is there anything we should change or stop doing?

### Data Analysis

The analysis was conducted in NVivo 12 Pro. The first (RHB) and last (AMSS) author performed the analysis. Both authors were part of the project team that had conducted the LOCI strategy. Both authors read the interviews. The coding and conceptualization of codes into themes were performed by the first author in close collaboration with the last author. Any disagreements in the interpretation of the material were resolved through discussion until consensus was reached.

Data were analyzed based on Braun and Clarke’s (2006, 2020) reflexive approach to thematic analysis with their conceptual model of six analytic phases as a starting point. Choosing this approach was based on our desire to identify patterns in the leaders’ descriptions of how they had experienced participating in LOCI. We approached the material inductively and semantically, meaning that coding and theme development were directed by and reflected the explicit content of the data. The first and the last author read all interviews and noted features relevant for the research question using memos in Nvivo. We expected to find differences in how the leaders had experienced participating based on our sampling strategy (i.e., high and low engagement). Interestingly, no clear-cut differences between the two groups were evident. Thus, this aspect became less important for our reading and analysis of the data than expected.

After thoroughly reading all the interviews, the first author systematically coded the data. Although data was coded without any predefined theoretical concepts (i.e., inductively rather than deductively), the research question guided the coding towards instances relevant to leaders’ experiences of LOCI participation. Descriptions of positive and negative aspects of participating differed in both specificity and comprehensiveness during each interview and between leaders. This variability is also reflected in the final collection of codes. A sample of codes under each theme can be found in Table 2.

**Table 2:** Overview of themes, prevalent codes, and theme definitions

Theme	Code examples	Theme definition
Structuring the EBP implementation	Taking stock Reminder of the implementation Prioritizing issues Bringing implementation forward Making plans and following them	LOCI helped participants plan, organize, and execute the EBP implementation
Taking responsibility for the EBP implementation	Creating new routines and systems Utilizing existing routines and systems Facilitating for therapists Encouraging therapists Involving the whole clinic	LOCI prompted participants to involve themselves in the EBP implementation in ways they otherwise would not have done
Interacting with others about EBP implementation	Preparing for meetings Reflecting after meetings Learning from others Receiving advice Sharing challenges	LOCI provided participants with opportunities to interact with others about the EBP implementation
Becoming aware of EBP implementation and their own leadership	What EBP implementation entails Own role in EBP implementation Own leadership role beyond EBP implementation Identifying areas for development Receiving feedback	LOCI raised participants’ awareness of EBP implementation—what it entails and own role in it—and leadership beyond EBP implementation

After having systematically coded all interviews, we continued to theme development. We followed Braun and Clarke’s definition of themes as “patterns of shared meaning, united by a central concept or idea” (Braun & Clarke, 2020, p. 14). This is opposed to an understanding of themes as simply data topics or domains, which is common in other approaches to thematic analysis. To generate initial themes, the first author reviewed all the codes and collated data by similarities and overlap between codes. The codes grouped around several clusters that we identified as candidate themes. When moving from these candidate themes to final themes, the first author iteratively reviewed each theme against included codes and other themes to ensure that all themes were themes in their own right rather than subthemes of another. In addition to working on theme generation, we noted codes that contradicted the overall patterns in the material. For a description of candidate themes and the process of moving from candidate themes to final themes, please see the Results section. When refining, defining, and naming themes, we aimed for precise and meaningful names and formulated short summary definitions for each theme. The first and last author also reviewed the themes one last time to ensure that they gave a sound representation of the patterns in the data.

## Results

### Candidate Themes

We initially identified several candidate themes that were later revised to form final themes. One candidate theme covered how the leaders described having practically dealt with the EBP implementation and how the LOCI strategy seemed to have helped with that. Codes in this candidate theme were diverse in content and scope (ranging from “Reminder of the implementation” to “Involving the whole clinic in the implementation”). This candidate theme was later split into two final themes with more distinct and clearly defined content. Other candidate themes included how the LOCI strategy functioned as community and support for the leaders, and several themes related to knowledge and awareness of leadership, roles, and EBP implementation. When later reviewing the latter group of themes, we concluded that these themes were better understood as subthemes of an overarching theme related to leaders’ awareness of EBP implementation and own leadership.

### Final Themes

The thematic analysis generated four major themes related to leaders’ experiences of participating in the LOCI strategy: (1) structuring the EBP implementation, (2) taking responsibility for the EBP implementation, (3) interacting with others about the EBP implementation, and (4) becoming aware of EBP implementation and their own leadership (see Table 2 for overview).

### “To Keep a Finger on the Pulse”: Structuring the EBP Implementation

This theme focuses on how LOCI’s structured format helped participants when planning, organizing, and executing the EBP implementation in their clinic. In particular, the regular consultation calls with the LOCI coach reminded the participants to focus on the EBP implementation and take stock: “The fact that I knew that on Tuesday at ten o’clock I’d have a short talk [with the LOCI coach]. Again, I gathered my thoughts around ‘where am I now with regards to this implementation’” (leader 1). The calls also worked as a general reminder of the EBP implementation in a work environment pressed for time: “The push, yes, those reminders. Everything, big and small, can drown out in the crowd and therefore being reminded and pushed forward has been the most useful” (leader 2).

Many participants found the combination of 360-degree assessment feedback reports, leadership development plans, and consultation calls particularly useful for advancing EBP implementation. The feedback reports and the leadership development plans helped them identify and prioritize issues that needed attention, and the consultation calls helped them stay focused on these issues over time. Leader 3 talked about how he worked together with his LOCI coach with the leadership development plan:

We’ve made a development plan for areas important for implementing [the EBP] in our clinic. (…) I feel the plan has become clearer and more tangible. So that’s been useful. And the other side of this is that the coach asked about progress and ‘where are you now concerning the plan you have made and how is the implementation going’.

Working on a plan with specific goals prompted the participants to focus on a few activities, which seemed to make the EBP implementation more manageable in a sometimes challenging work environment: “Often when you’re so tired in the clinic, the fact that I got help, when we worked on the plan you picked two to three things, ‘that’s what I’ll focus on’. (…) So it was very concrete on what I should do until next time” (leader 4).

A few participants expressed some ambivalence towards “the amount of effort we were expected to put in compared to other things” (leader 5). Leader 2 felt that the program was too comprehensive compared to what he gained: “The consultations are so frequent that hardly anything happens from one week to the next. Sometimes I have more frequent meetups with my coach than with all the therapists working with the treatment method, (…) and [it] is used with maybe five percent of the patients.” Most participants, however, found that, in the end, LOCI was worth spending time on. Leader 6 admitted:

I thought that this would be rather fussy, actually. (…) Then I thought it’s probably a way to keep a finger on the pulse. But I thought ‘do I really have time for this’. (…) Then I thought it was worth it because I find it bothersome not to have competent trauma therapists when so many [patients] get referred for that reason.

Thus, overall, the LOCI strategy’s structuring elements (e.g., feedback reports, leadership development plans, regular consultation calls) provided participants with concrete tools to use in their day-to-day management of the EBP implementation.

### “Defining a Direction and Showing the Way”: Taking Responsibility for the EBP Implementation

This theme describes how LOCI prompted the participants to involve themselves in the EBP implementation in ways they otherwise would not have done. LOCI motivated participants to support and facilitate for therapists in EBP training, for example by allocating time for them to learn and work with the EBP. Leader 7 recalled being actively involved in the therapists’ effort to work with the EBP: “What’s going on? (…) What do you need? I was very explicit on giving time and saying, ‘do you have patients’, ‘this is someone you can discuss [your case] with, make contact, they’re ready’”. LOCI also seemed to have prompted the participants to widen their scope. Leader 8 talked about how LOCI made him “more explicit about visions and goals, (…) trying to involve more people than just those few who participate in the project.” In fact, activities to ensure that everyone “is involved and understand that we’re pursuing this” (leader 3) was one of the key takeaways for most of the participants. Leader 9 described in more detail:

I was pretty excited about approaching transformational leadership. (…) About idealized influence. I wanted them [the therapists] to experience having an engaged leader who wants something, defining a direction and showing the way. I did this, and also took the opportunity to inspire to the fact that we pull together [in this implementation].

Another example is leader 6, who included the EBP implementation in the weekly meeting with the therapists as a low-threshold way to involve the entire clinic: “Every Tuesday morning we stop and look at the flow of things. And now I’ve included [the EBP] as a separate point.”

Several participants also referred to specific structural changes they had made based on LOCI, often involving minor tweaks in existing routines to support the EBP implementation. Leader 2, for example, said: “We’ve always had one of the TF-CBT therapists in the intake team, so they’ve said ‘hey, here’s a potential trauma case, we should give this to that therapist. Now that therapist needs a new patient’”. Leader 1 described how LOCI prompted her clinic to work more systematically with trauma screening: “We work a lot with trauma in this clinic, (…) but a tool to investigate how many who needs treatment for posttraumatic stress during a three-month period has been very interesting to use systematically.”

Although all participants acknowledged the LOCI strategy’s potential usefulness and relevance for EBP implementation, two of the participants seemed unsure whether LOCI had prompted them take more responsibility for this EBP implementation than they otherwise would have done. Interestingly, their reasons for feeling this way were quite different. Leader 2 expressed that his therapists in EBP training were so self-sufficient and proficient from the start that the implementation “would’ve succeeded anyway”, elaborating: “What I’ve chosen as leadership method or approach, I would’ve done anyway, (…) so it’s only been a confirmation of what would’ve happened anyway”. For leader 5, on the other hand, the situation was the complete opposite; the EBP implementation had gotten off to a bad start with “very little information and codetermination” from upper-level management, combined with key employees having pre-existing reservations against the EBPs. She explained:

There was so much conflict in the workplace and a lot of discontent with the management and the project, which made me feel I couldn’t just push on with the implementation as we otherwise would have. (…) It felt like there was no point because the starting point was so bad. I didn’t feel that I had a chance to make this work in a good way anyway.

Although she appreciated the support from her coach and fellow leaders during LOCI participation, the bad start for the EBP implementation combined with a challenging work environment seemed to have made it difficult to follow through with the LOCI components once resistance to the EBP implementation had spread among the therapists.

### “Entering a Bubble”: Interacting with Others About the EBP Implementation

This theme is about how the LOCI strategy provided participants with opportunities to interact with others about the EBP implementation. Highlighted by all the participants was the sense of community they had experienced during the quarterly workshops, “a bit like entering a bubble” (leader 2). During the workshops, the participants got a chance to “exchange experiences and ideas (…) and pick up things to try out back home” (leader 6). Differences in educational and personal backgrounds seemed to have enhanced this experience: “We complemented each other. We could reflect upon the same thing (…) from different angles based on our personalities or professional viewpoints” (leader 10). The workshops provided a safe space for participants to share challenges and receive support. Leader 11 recalled:

Most important for me at the very beginning was getting confirmed that it’s difficult everywhere. It wasn’t just me who had particularly difficult circumstances or particularly difficult therapists, they were everywhere. (…) It wasn’t like all about showing off, but about being honest about the challenges you faced.

In the weekly consultation calls with the LOCI coach, the participants could discuss challenges related to the EBP implementation and their own leadership with an outside person. Many participants found this useful: “Even if [the coach] didn’t always bring anything new to the table, I had someone to discuss with and could think out loud” (leader 9). The consultation calls also helped some participants get through particularly challenging stages in the implementation. Leader 4 described having struggled with her own motivation for the EBP implementation and her coach helping her gain momentum: “Recognizing that there are difficult stages and that everything doesn’t run smoothly. That helped for a sort of perseverance, because I resigned a little bit at one point. (…) [The coach] didn’t actually use the word ‘get a grip’, but it was a couple of those… so I kept my chin up”.

For some, LOCI participation also fostered internal cooperation with other first-level leaders and across leadership levels within the same health trust. Leader 1, for example, described how she and another first-level leader in the same organization got “allocated time for leadership” between meetings: “We’re two people participating together, and that made us think ‘today it’s the group call, where are we now’ (…) and afterwards figuring out, ‘okay, in [another clinic] they’ve done this differently than us’ and ‘is it useful for us to think this way’”. Thus, they prepared for and reflected upon topics raised during workshops and group consultation calls together, which seemed to enhance their overall involvement with each other and with the EBP implementation.

### “A Self-insight Boost”: Becoming Aware of EBP Implementation and Their Own Leadership

This theme reflects how the LOCI strategy raised participants’ awareness of EBP implementation—what it entails and their role in it—and their leadership behavior beyond EBP implementation. Many participants admitted having started LOCI viewing it as “part of the deal” (leader 7) to gain access to EBP training. For most participants, however, their impression changed, and with it came a recognition of the importance of implementation strategies per se. This realization was mainly reflected in how the participants contrasted past experiences of implementing new practices with experiences following the LOCI strategy:

Some years ago, we participated in another implementation project. However, it was not implemented this way. It only included training people in [name of EPB]. We see that the competence we built at that time is more or less gone. It seeps out, people take new courses, find other jobs. In a way, it’s not integrated into the organization. (leader 6)

The consultation calls were highlighted by many participants as important for strengthening awareness of their own behavior and the crucial role leaders play in EBP implementation, particularly through the continuous focus on linking concrete leadership behaviors to implementation challenges at different stages in the implementation. Leader 4 described:

My coach followed us where we were in the implementation process and I got input on perspectives on how to work with the whole implementation process: ‘Okay, now this is your challenge, then we need to talk about individual consideration one more time.’ If, for example, resistance among the therapists was the issue, I got to work with that.

Several participants also talked about how LOCI had made a lasting impact on the priority they put on EBPs far beyond this specific project. Leader 9, for example, described her new perspective on staff recruitment: “It’s a clear change in my level of awareness when I’m hiring. I give very clear expectations: if you are to work here, you must reckon with the fact that this is an evidence-based workplace. You must expect readjusting and lifelong learning through evidence-supported educations.”

LOCI also offered participants opportunities to develop themselves as leaders beyond learning new implementation leadership skills. Many participants referred to having had eye-opening experiences when presented with the results from the 360-degree assessments. Leader 11 recalled:

To involve the employees in such a way that they both understand and feel included. I was much worse at this than I thought. I learned this through LOCI when I received feedback from the 360-reports and the consultations. My self-esteem didn’t exactly rupture, but I got a self-insight boost that I’m eternally grateful for.

The participant’s use of the phrase “self-insight boost” illustrates how getting evaluated and receiving unexpected feedback was a largely positive experience. A couple of participants, however, expressed reservations towards the feedback reports and how they were used in the program. Although none of them found the process outright uncomfortable, they nevertheless felt that the reports could not be trusted. Leader 2 elaborated:

I don’t think the assessments has been useful. I feel that you’re trying to interpret some numbers (…) and I think that the employees found these questions difficult to answer, especially those who were not part of the EBP training, so they probably answered a bit at random, and when discussing what the changes in these assessments could mean, I was like, ‘yes ok, I’ll listen and I’ll try to nod and bow whenever appropriate’.

Although several others agreed that the questions used in the 360-degree assessments could benefit from some adaptation to the specific context, the large majority of participants described the process of receiving concrete and specific feedback positively; the feedback reports prompted curiosity and reflection and provided valuable insights into their own leadership role and the EBP implementation.

## Discussion

Successful and sustainable EBP implementation is important on a health care system level as well as from clinic, clinician, and patient perspectives. EBP training alone is not sufficient to achieve practice change (Beidas & Kendall, 2010). The literature highlights systematic use of implementation strategies adapted to the specific context as important for achieving implementation goals (Powell et al., 2015). First-level leaders play an important role in EBP implementation due to their close and frequent contact with practitioners (Aarons et al., 2017a, 2017b). Thus, particular attention should be given to preparing and training them for this role. Among the 73 discrete implementation strategies identified by the Expert Recommendations for Implementing Change (ERIC), one is devoted to leadership training to foster and support effective leadership of EBP implementation (i.e., “Recruit, designate, and train for leadership”) (Powell et al., 2015). The LOCI strategy is a multifaceted implementation strategy directed towards first-level leaders simultaneously recognizing the importance of organizational climate and context. Our thematic analysis generated four themes related to first-level leaders’ experience participating in this strategy and points to some key ways in which the LOCI strategy can help first-level leaders in their endeavor to support EBP implementation and improve quality of care.

First, implementation strategies such as the LOCI strategy can make it easier for first-level leaders to structure the EBP implementation in their clinic. Specifically, participants in our study conveyed that the regular meeting points and data-driven leadership plans served as reminders and helped prioritize issues related to EBP implementation in a work environment with high time demands. This is important considering previous findings indicating that time constraints can serve as barriers to practice change (Williams et al., 2015). Implementation strategies such as the LOCI strategy can also support first-level leaders to take responsibility for the EBP implementation in more substantial ways (Richter et al., 2016). Specifically, participants in our study said they prioritized the EBP implementation to a larger extent than they otherwise would have, for example by not only motivating the practitioners who received EBP training, but also including the whole clinic in their vision to implement EBPs for PTSD. Both examples are central to transformational leadership (Bass & Avolio, 1990) and highlighted by mental health practitioners as important for sustaining EBPs (Egeland, 2019a, 2019b). The latter is also in line with an identified ERIC implementation strategy related to mandating change through having “leadership declare the priority of the innovation and their determination to have it implemented” (Powell et al., 2015). Participants also found ways to adjust existing routines to facilitate for the EBP implementation. One of the key challenges with implementing change is sustainment over time (Wiltsey Stirman et al., 2012), and using established routines might make new practices more likely to be sustained beyond active implementation (May & Finch, 2009).

Furthermore, interventions such as the LOCI strategy can provide first-level leaders with valuable support and additional learning opportunities during the challenging journey of EBP implementation. Notably, all participants in our study spoke at length about the support they received through the LOCI strategy, both from other leaders and from their LOCI coach. This kind of community is not always easy to come by for people in leadership roles who are not exempt from feelings of loneliness (Silard & Wright, 2020). Importantly, several leaders emphasized the value of this support in its own right, but also as a means to an end. Particularly when challenged, participants appreciated sharing their difficulties with peers and with an outside person represented by the LOCI coach. In this way, they could receive advice and at the same time learn from their own and others’ experiences. Additionally, the mutual sharing among the leaders gave many participants a sense of community and perseverance in common challenges related to the EBP implementation and leadership in general.

Finally, the LOCI strategy focuses on what has arguably been an important goal of dissemination and implementation initiatives, namely increasing awareness of the importance of EBP implementation in practice (Aarons et al., 2014a, 2014b, 2017a, 2017b). Specifically, participants in our study described how participating in the LOCI strategy made them realize the importance of EBP implementation and the role they as leaders play in facilitating and realizing practice change. Furthermore, they also seemed to have developed themselves as leaders in general, by becoming more aware of their own leadership behaviors and how they are perceived by their employees. The last point is particularly note-worthy given literature indicating that leaders who are in tune with their followers (i.e., self-other agreement) achieve better results (Fleenor et al., 2010), including in mental health services (Aarons et al., 2017a, 2017b).

In sum, the features evident from our thematic analysis speak to one overall lesson shared among the participants, namely, that change does not happen by itself. This lesson appeared to have occurred as a function of two parallel learning processes—or mechanisms—operating during LOCI participation: First, an *awareness-raising* process in which the participants came to realize that practice change requires effort by themselves and others, and second, an *enabling* process, in which they acquired specific tools and gained access to support for making this change happen. This change in mindset draws attention to the complexity of leadership skill development, and supports the need for evaluations to use in-depth qualitative research to capture these intangible training outcomes of both implementation strategies focused on implementation leadership and general leadership development programs (King & Nesbit, 2015). A qualitative methodology allowed the participants to retrospectively reflect upon what they had experienced and gained from participating in the LOCI strategy beyond immediate reactions and measurable results. Strikingly, several participants spoke of how they would use these newly acquired resources in future projects, or how they would have used them in past implementations gone wrong. This is interesting, as it indicates the potential for implementation strategies to build competence lasting beyond the specific implementation project in which they are used.

Moving on to some of the less positive experiences conveyed through our analysis, two participants in particular had reservations about the LOCI strategy’s benefits for the EBP implementation in their clinics. Their narratives point to important challenges for implementation science to address. Specifically, our analysis revealed that the participants’ reservations were based on two very different situations related to perceptions of organizational context and readiness: one experienced the EBP implementation as (almost) too easy, and the other felt it was too hard to even give it a try. In the former case, the practitioners in EBP training were seemingly so self-sufficient and proficient that the leader concluded the implementation would have succeeded both with and without help from the LOCI strategy. Although we cannot say based on our data whether the leader’s perception was accurate, this raises the question of whether an implementation strategy focused on implementation leadership is a good investment for clinics where leadership is strong and a supportive climate for EBP implementation already exists. On the other hand, overconfidence can have deleterious consequences for leadership effectiveness (Shipman & Mumford, 2011). Thus, a key challenge for implementation strategies is to identify and engage leaders who perceive few challenges—and therefore find implementation leadership redundant—but where their organization actually lacks a supportive climate for EBP implementation.

In the latter case, the participant conveyed organizational challenges that overshadowed and hampered the EBP implementation from the start. As such, low organizational readiness for change (Weiner, 2009) seemingly limited the agenda for what was possible to achieve even with the support of an implementation strategy. The LOCI strategy is designed to primarily support EBP implementation, and this leader’s narrative illustrates how the intervention might not be as helpful for leaders in organizations with a negative molar climate (Williams et al., 2018). Thus, these organizations might need to address other issues first before they are ready to start EBP implementation. Drawing upon the Exploration, Preparation, Implementation, Sustainment (EPIS) framework (Aarons et al., 2011), implementation strategies may want to focus more on the early phases of EBP implementation (i.e., Exploration and Preparation). In the current implementation trial, information meetings with the leaders and contracts specifying mutual requirements were used to ensure buy-in before inclusion (Egeland, Hauge, et al., 2019; Egeland, Skar, et al., 2019). Future implementation projects could benefit from going one step further by measuring organizational readiness to change and intervening before implementation to ensure the organization is ready for EBP implementation and to prevent undetected organizational challenges from hampering the implementation.

These results echo those from studies in the leadership development literature; context and individual characteristics such as work environment and motivation are often central for understanding training transfer (e.g., Velada et al., 2007). The LOCI strategy has a strong theoretical base in the full-range leadership model (Bass & Avolio, 1990) and the implementation leadership literature (Aarons et al., 2015), and consists of effective components supported by research (e.g., 360-degree feedback (Lacerenza et al., 2017)). Nevertheless, the two narratives above illustrate how context and individual characteristics interact with intervention design and delivery to produce differing learning experiences and outcomes. Thus, our results provide valuable insights into possible boundary conditions of the LOCI strategy (i.e., when is it most beneficial, when is it not as beneficial). The strategy seemed to have worked well for most participants in our study and previous research also indicates that it is an effective implementation strategy overall (Aarons et al., 2015; Skar et al., 2022). However, it might not be as beneficial for extreme cases. On the one extreme, when implementation climate already is strong with little room for improvement, the LOCI strategy might not be worth the time and effort demanded from the leaders to participate in full. On the other extreme, when organizations face organizational challenges outside the scope of an implementation strategy focused on implementation leadership and strategic implementation climate, they might need other interventions to address issues such as overall molar climate or organizational trust. The LOCI strategy is resource-intensive for both LOCI providers and participants, and further research into the conditions for when the strategy is effective and when it is not is highly needed.

Some precautions should be taken when interpreting the results, as all knowledge is situated (Madill et al., 2000). First, we as researchers did not approach the data without assumptions. The research question guided our analysis towards instances relevant to shed light on leaders’ experiences of participating in the LOCI strategy. The fact that we had experience working with the strategy may also have influenced how we read, coded, and interpreted the data (e.g., knowing the theoretical background of the LOCI strategy, having met the participants during workshops). When recruiting participants, we aimed at capturing a breadth in experiences with our purposeful sampling of leaders with both high and low engagement rates. In the end, however, we did not see any clear-cut differences in experiences between the two groups. Most participants talked about the benefits (rather than the downsides) of LOCI participation. Furthermore, although the interviewers were not formally part of the LOCI team, the participants may have seen them as representatives of the project, which in turn might have influenced what they chose to convey during the interviews (e.g., under-communicating criticism of the strategy due to social desirability). Thus, we might not have succeeded in capturing the whole range of positive and negative experiences of participation. Given our sample size, there is also a chance that data from more participants would have generated larger variability. On the other hand, relatively little variability and the clustering of codes around a few key themes related to benefits might indicate that saturation of themes was reached.

We should also take into account how contextual factors might have shaped the leaders’ experiences of LOCI participation during this particular implementation. Importantly, clinics volunteered to participate in the implementation project, and leadership at all participating clinics were positive towards implementing EBPs for PTSD and saw this as a good opportunity for service improvement at their clinic. This likely gave most leaders a favorable view of participating in an intervention to enhance implementation success. Furthermore, the national implementation was conducted on behalf of the Norwegian Directorate of Health and financing was secured via the national budget. Thus, the project took place in an enabling and predictable outer context compared to many other implementation projects.

## Conclusion

Our in-depth qualitative analysis of first-level leaders’ experiences of participating in the LOCI strategy suggests that it helped the majority of participants in essential ways when implementing EBPs for PTSD in their clinic, and inspired and motivated action when they encountered challenges. Specifically, the LOCI strategy raised participants’ awareness of their role as leaders in bringing about practice change and provided tools and support for them to manage and lead the EBP implementation in practice. This underscores the importance of implementation strategies aimed at first-level leaders as a way to engage and empower them to get involved in implementation processes. Furthermore, participants seemed to have gained awareness and competencies perceived as useful beyond this particular implementation. Thus, building and sustaining implementation leadership through implementation strategies such as the LOCI strategy might be a fruitful way to increase the potential and readiness for practice change in mental health services in general.

## Data Availability

The data material in anonymized format is available from the corresponding author upon reasonable request.

## Abbreviations

LOCI: Leadership and Organizational Change for Implementation
EBP: Evidence-based practice
PTSD: Posttraumatic stress disorder

## Declarations

### Standards of Reporting

When preparing this manuscript, authors followed the Standards for Reporting Qualitative Research (SRQR) hosted by the EQUATOR Network.
